# Multimodal AI diagnostic system for neuromyelitis optica based on ultrawide-field fundus photography

**DOI:** 10.3389/fmed.2025.1555380

**Published:** 2025-05-07

**Authors:** Simin Gu, Tiancheng Bao, Tao Wang, Qiting Yuan, Weiguang Yu, Jiayi Lin, Haocheng Zhu, Shihai Cui, Yi Sun, Xiuhua Jia, Lina Huang, Shiqi Ling

**Affiliations:** ^1^Department of Ophthalmology, The Third Affiliated Hospital, Sun Yat-Sen University, Guangzhou, China; ^2^Zeemo Technology Company Limited, Shenzhen, China; ^3^Shenzhen Eye Hospital, Jinan University, Shenzhen, China

**Keywords:** neuromyelitis optic, neuromyelitis optica spectrum disorders, deep learning, artificial intelligence, diagnostic model

## Abstract

**Purpose:**

While deep learning (DL) has demonstrated significant utility in ocular diseases, no clinically validated algorithm currently exists for diagnosing neuromyelitis optica (NMO). This study aimed to develop a proof-of-concept multimodal artificial intelligence (AI) diagnostic model that synergistically integrates ultrawide field fundus photographs (UWFs) with clinical examination data for predicting the onset and stage of suspected NMO.

**Methods:**

The study utilized the UWFs of 330 eyes from 285 NMO patients and 1,288 eyes from 770 non-NMO participants, along with clinical examination reports, to develop an AI model for predicting the onset or stage of suspected NMO. The performance of the AI model was evaluated based on the area under the receiver operating characteristic curve (AUC), sensitivity, and specificity.

**Results:**

The multimodal AI diagnostic model achieved an AUC of 0.9923, a maximum Youden index of 0.9389, a sensitivity of 97.0% and a specificity of 96.9% in predicting the prevalence of NMO on test data set.

**Conclusion:**

Our study demonstrates the feasibility of DL algorithms in diagnosing and predicting of NMO.

## 1 Introduction

Neuromyelitis optica (NMO), also known as Devic syndrome, is an idiopathic neuroinflammatory disorder. This rare relapsing clinical syndrome characterized by astrocytopathy of the central nervous system, with a predilection for the optic nerves and spinal cord (1), disease occurs globally and affects individuals of all ethnicities (2). Its primarily manifestations include acute optic neuritis (ON) (3) and transverse myelitis (TM) (4), both of which cause blindness and paralysis. NMO has a poor prognosis and has long been considered a clinical variant of multiple sclerosis (MS). However, the disease is now studied as a prototypic autoimmune disorder on the basis of the discovery of a novel and pathogenic anti-astrocytic serum autoantibody that targets aquaporin-4 (AQP4-Ab) and IgG autoantibodies to myelin oligodendrocyte glycoprotein (MOG-IgG). Pathogenetic AQP4-IgG is responsible for more than 80% of NMO cases (5), while approximately 10–40% of individuals with NMO lack AQP4-IgG and instead exhibit pathogenetic MOG-IgG (6). Although the AQP4 antibody is highly specific for this disorder, some patients harboring this antibody may present with isolated ON or TM. Magnetic resonance imaging (MRI) is commonly used to identify and characterize lesions in suspected NMO cases, helping to distinguish between NMO and MS. To encompass the broader clinical spectrum, including atypical or incomplete presentations, the term ‘neuromyelitis optica spectrum disorders’ (NMOSD) was subsequently introduced to refer to NMO and its formes frustes (7).

A subset of patients with NMO exhibit a variety of different symptoms indicating brain or brainstem involvement; however, ON and TM are the predominant manifestations of the disease. Ocular complications frequently include reduced visual acuity, a decline in high-contrast visual acuity, color desaturation (8), scotoma, and ocular pain during eye movement (9). These visual disturbances frequently serve as critical diagnostic indicators of NMO. If left untreated, NMO can lead to severe and irreversible visual impairment as well as significant motor dysfunction owing to incomplete recovery from acute attacks. Hence, advancements in early and accurate diagnosis are crucial, as they would facilitate prompt therapeutic intervention and significantly improve long-term clinical outcomes for patients with NMO.

Currently, the clinical diagnosis of NMO is conducted by qualified ophthalmologists or neurologists based on clinical presentation, disease course, and the detection of specific autoantibodies, supplemented by MRI scanning (10). Notably, the confirmation process requires specialized clinical expertise, posing a difficult challenge in resource-limited settings such as developing countries or rural communities where access to trained ophthalmologists or neurologists is scarce. Taken together, existing approaches for detecting NMO have substantial limitations in terms of accessibility and widespread implementation. Given the increasing global health burden associated with this disease, there is an urgent need to develop innovative diagnostic strategies that can overcome these constraints, enhance early detection, and ultimately improve patient outcomes.

Advancements in artificial intelligence (AI), particularly in machine learning (ML) and deep learning (DL), has driven remarkable breakthroughs in different areas of research (11–13). In ophthalmology, the widespread availability of fundus digital imaging has sparked growing interest in leveraging AI for early diagnosis and treatment in retinal and optic nerve disorders (14). Among these imaging modalities, ultrawide field fundus photographs (UWFs) have gained prominence in medical applications, particularly for ocular disease detection. UWFs offers various merits, including their suitability for nondilated pupils, the speed of acquisition, and the wide retinal imaging ranges (up to 200°) (15). Consequently, a variety of studies have explored the integration of AI with UWFs for diagnosing and treating of various ocular conditions such as diabetic retinopathy (16), age-related macular degeneration (17), cataracts (18), glaucoma (19, 20) and myopia (21). DL methodologies based on UWFs have become instrumental in reshaping diagnostic strategies in ophthalmology. Notably, DL methodologies based on UWFs have emerged as transformative tools, reshaping diagnostic paradigms in ophthalmology and paving the way for more efficient, accurate, and accessible disease detection strategies.

Standard color fundus photographs provides a 30 to 50-degree image whereas UWFs provide an encompassing view of the retina, allowing examination of not only the central retinal area but also the peripheral zones, which able to detect predominantly peripheral lesions in eyes with its wide coverage (22). The analysis of ultrawide field fundus could be of value in screening, given the prognostic importance of peripheral lesions in distinguishing NMOSD from other fundus disease progression. However, despite NMOSD being a major cause of visual impairment worldwide, the application of AI in its diagnosis remains relatively unexplored. This raises an intriguing question: could NMOSD be accurately diagnosed solely through UWF imaging, without the need for multiple diagnostic modalities? Exploring this possibility could pave the way for more accessible and efficient diagnostic strategies in clinical practice.

## 2 Methods

### 2.1 Subject characteristics

This clinical study adhered to the tenets of the Declaration of Helsinki and was approved by the research ethics committee of the Third Affiliated Hospital of Sun Yat-sen University.

The study utilized the UWFs of 330 eyes from 285 NMO patients and 1,288 eyes from 770 non-NMO participants, along with clinical examination reports were initially selected for the study. All the enrolled patients were referred to the Third Affiliated Hospital of Sun Yat-sen University from January 2022 to April 2024. The whole process was performed at the hospital, and informed consent was obtained from all study participants. The inclusion criteria included a diagnosis of NMO confirmed by a qualified neurosurgeon from the Third Affiliated Hospital of Sun Yat-sen University, based on diagnostic criteria established in previous population-based studies. Patients excluded from the possibility of NMO were classified as non-NMO, however, they could present with other ocular pathologies, such as diabetic retinopathy, glaucoma, retinal detachment, or retinal degeneration.

### 2.2 Multimodal dataset preparation

**Figure 1:**
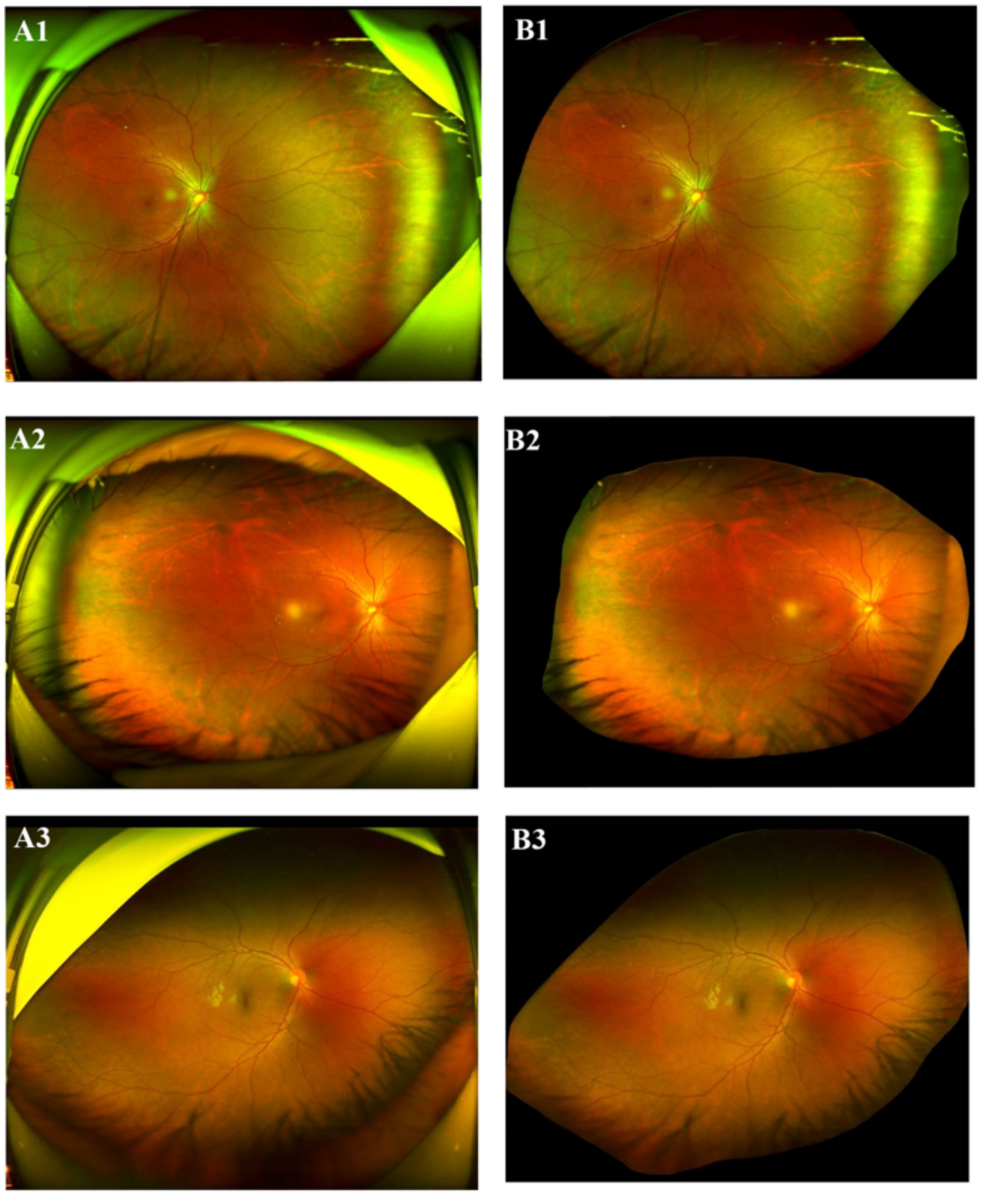
Original SLO image (LHS) and processed image (RHS). **(A1–A3)** Original SLO image; **(B1–B3)** excessive “image border”removed image.

The dataset used in this study contains the data from two modalities: UWF fundus images, captured using scanning laser ophthalmoscopy (SLO, Optos Daytona) and clinical examination reports. To ensure the quality of the research, stringent image quality control criteria were implemented. All images were captured by extensively trained and specialized technicians, ensuring that the scanning quality of each image adheres to the standards of clarity and visual interpretability, absented from significant motion artifacts, and with precise centration on the optic nerve head or macula. Images failing to meet these criteria were excluded. During data preprocessing, all images were assessed for quality using both automated algorithms and manual inspection by experienced ophthalmologists. Images with severe artifacts that could compromise feature extraction were excluded from analysis. All UWFs analyzed in the present study were restricted to a single, highest-quality capture per patient, as determined by standardized ophthalmic imaging quality metrics (focus clarity ≥70%, illumination uniformity, and absence of artifacts). Six ophthalmologists with clinical experience were recruited to evaluate the images and clinical examination reports. To ensure privacy, all images were first deidentified to remove any patient-related information. Subsequently, the images were preprocessed using a pretrained deep learning segmentation model to remove extraneous “image borders,” including the eyelids and the edges of the scanning machine, which are irrelevant to the current classification task. The segmentation model removes these image borders by generating a predicted mask of the detected border region while retaining the retina region. The output image of the segmentation model is padded using black pixels to produce a square shape with the retina area centered vertically for model input. This approach is well-justified, particularly in tasks that prioritize critical retinal regions, such as the optic disc and macula. By employing this method, interference such as eyelids, and machine edges can be minimized, data quality can be significantly improved, and overall model performance can be enhanced. This represents a widely accepted and validated practice in the field. Figure 1 shows the original SLO image and the corresponding preprocessed “borderless” image.

**Figure 2:**
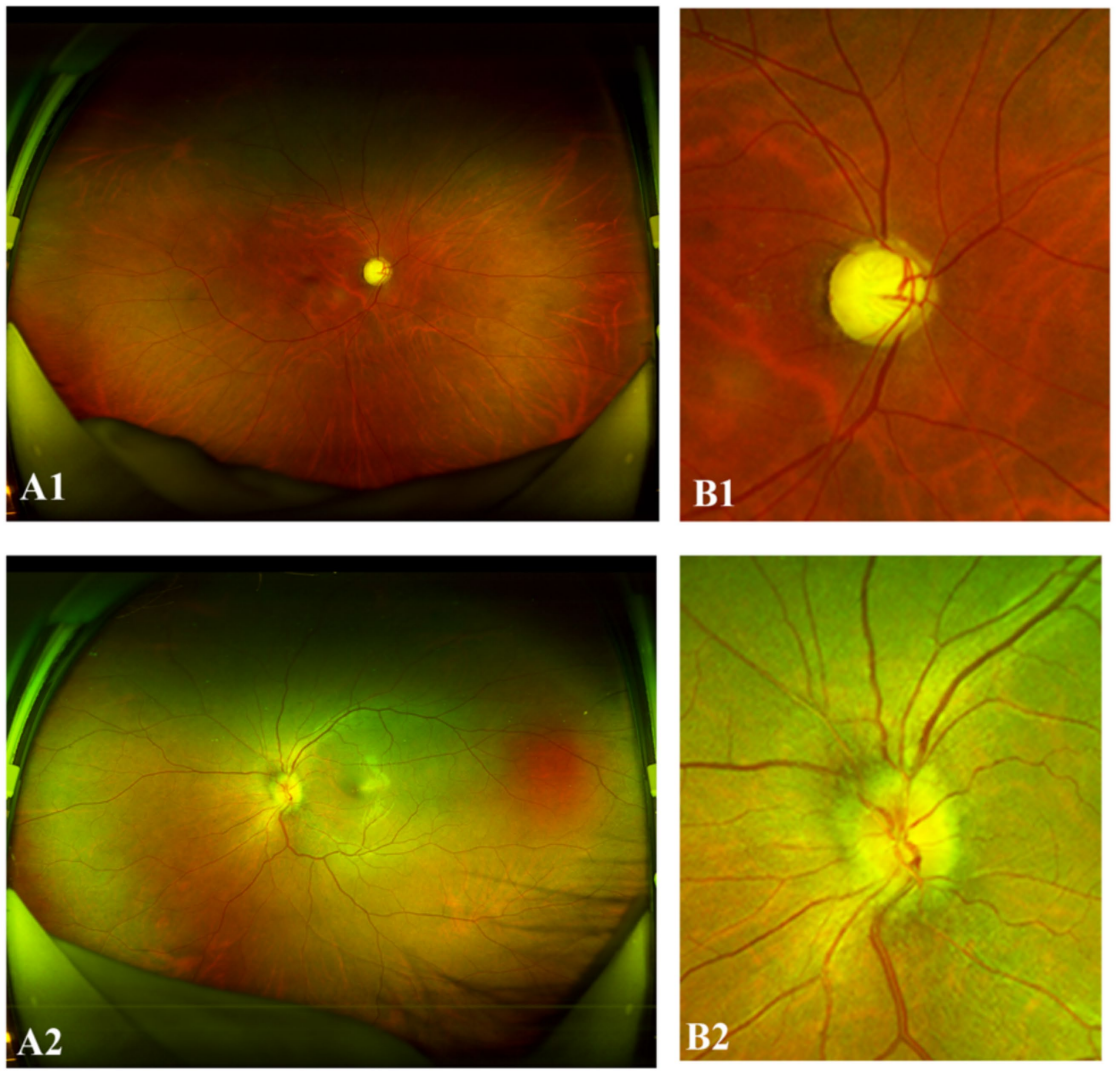
Representative “positive” NMO UWF images. **(A1,B1)** The right eye of a 34-year-old female with NMO demonstrated a partially increased cup-to-disc (C/D) ratio. **(A2,B2)** The left eye of a 31-year-old female with NMO exhibited optic disc hyperemia, edema, and indistinct margins.

Three types of clinical examination reports, including those with data from MRI of the optic nerve, MRI of the spine and AQP4 antibody tests, were collected and analyzed. The model prediction probabilities and clinical data are integrated using a conditional decision approach. Specifically, the image score, obtained from the DL module, and the clinical score, derived from the clinical criteria, are weighted and input into the model to generate the final NMO prediction. The reports for the optic nerve MRI scans and the AQP4 antibody test were considered either positive or negative. Categorical variables were first converted into two dummy variables (1 for positive and 0 for negative) through manual coding, followed by one-hot encoding for model input. The record for spine MRI is an unstructured text description of the MRI result. Thus, the spine MRI results were preprocessed into dummy variables as follows: (a) the results were encoded as 1 (positive) if demyelinating disease was observed across three consecutive vertebrae; (b) the results were encoded as 0 (negative) if demyelinating disease was not observed or if it did not affect three consecutive vertebrae. Finally, we created a new dummy variable named “clinical result,” which was set to 1 if any of the dummy variables above was positive and 0 otherwise. The composite clinical outcome was coded as positive (1) if any one of the following criteria were present: AQP4-IgG seropositivity, orbital MRI demonstrating characteristic features diagnostic of NMO, or spinal cord MRI showing lesions spanning ≥3 vertebral segments. Certain cases exhibiting characteristic optic nerve abnormalities, including optic disc hyperemia, edema, indistinct margins, and a partially increased cup-to-disc (C/D) ratio suggestive of optic nerve atrophy (Figure 2) were classified as high-risk for NMO. For all 330 included fundus images, matched clinical data were available. Cases with missing data were classified as positive if at least one available criterion was met; if all criteria were missing, the AI prediction alone determined the outcome.

The image dataset comprised 1,618 images in total (330 or 20.4% NMO positive and 1,288 or 79.6% NMO negative), among which 330 were associated with clinical records. The dataset was split into training, validation and test datasets at a ratio of 80:10:10 on the basis of a stratified-group-split approach, i.e., stratification by NMO labels and grouping by subject ID.

### 2.3 Model design

**Figure 3:**
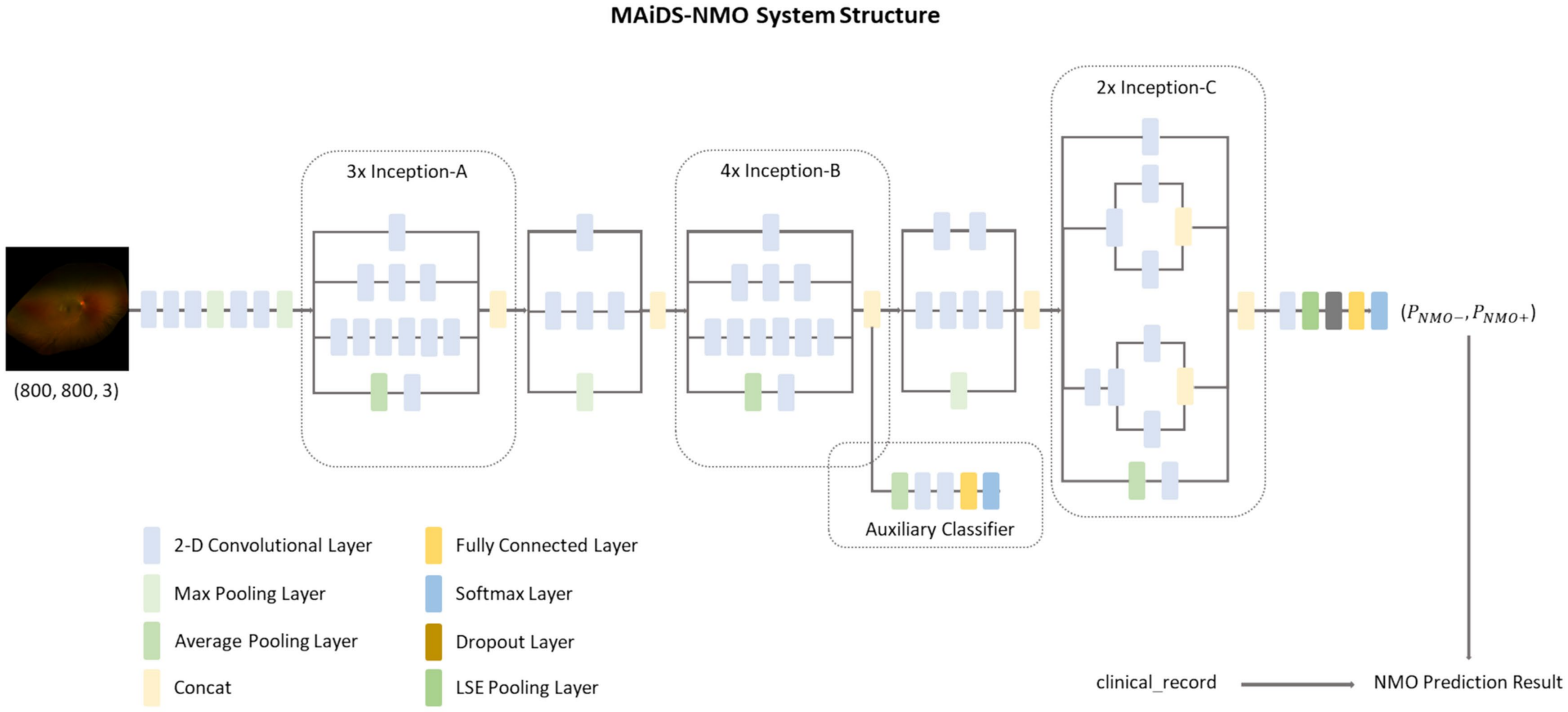
The MAiDS-NMO system structure. MAiDS-NMO takes two input modalities of fundus image and clinical result. The fundus image is then fed to a deep neural network model for NMO likelihood inference. Finally, the clinical result is used together with the predicted NMO probability (based on fundus image) to reach a positive or negative prediction of NMO.

We developed a multimodal AI diagnosis system for NMO (MAiDS-NMO), the structure of which is depicted in Figure 3. MAiDS-NMO takes inputs from two modalities: fundus imaging and clinical results. The fundus images are fed to a deep neural network model for NMO likelihood inference, and the clinical result is used together with the predicted NMO probability from the fundus images to make a positive or negative prediction of NMO. The deep model is Inception-V3 with Log-Sum-Exp (LSE) pooling (replacing the original adaptive average pooling method) (23), enabling pixels with similar scores to have similar weights in the pooling process during training.


$$\begin{array}{l} x_{LSE\_p}=\frac{1}{r}\log\left[\frac{1}{w*h}\underset{\left(i,j\right)}{\sum }rx_{ij}\right],\mathrm{where}\;r\;\mathrm{is}\;a\\ \mathrm{hyperparameter},\;\left(h,w\right)\mathrm{is}\;\mathrm{input}\;\mathrm{shape}\text{.} \end{array}$$

### 2.4 Model development

We use the PyTorch implementation of the Inception-V3 model (LSE pooling) with the pretrained weight file “inception_v3_google,” which was developed by Google using the ImageNet dataset (ILSVRC-2012-CLS dataset for image classification) under the TensorFlow framework. One NVIDIA GeForce RTX 3090 Ti (24 GB) GPU was used for model development.

The training dataset was used to fine-tune the ImageNet pretrained Inception-V3 model, while the validation dataset was used to select the best weights (those which yielded the minimum validation loss). We adopted a three-step “warm-up” procedure during training, i.e., we froze all the layers (blocks) except for the fully connected dense layer of the model for the first several epochs of training and gradually unfroze Block 7, then Blocks 5 & 6, and finally the remaining blocks of the model. This method effectively prevents the training process from being trapped by a local minimum before it finds a global minimum.

The diagnosis of NMO can be reliably established through serological antibody testing (AQP4-IgG/MOG-IgG) and characteristic neuroimaging findings on MRI. Our diagnostic model developed exclusively using UWFs parameters demonstrated comparable diagnostic accuracy (AUROC 0.97, specificity 0.92). After training the DL model with the dataset of 1,618 fundus photos, including 330 NMO images, our diagnostic model developed exclusively using UWFs parameters demonstrated high confidence in the results, with an area under the curve (AUC) > 0.97 and a specificity > 92% in distinguishing NMO from non-NMO fundus photos. Notably, the model incorporating comprehensive clinical metadata showed enhanced diagnostic performance, with an area under the curve (AUC) > 0.99 and a specificity > 96%, though comparative analysis revealed no statistically significant difference between the two models.

Five-fold cross-validation was performed to rigorously evaluate the robustness and generalizability of the model. This methodological approach entails partitioning the dataset into five equal subsets, wherein the model is iteratively trained on four subsets and validated on the remaining one. By systematically rotating the test set across all five folds, this technique facilitates a comprehensive assessment, mitigating potential biases associated with a single training-test split.

### 2.5 Comparative test

**Figure 4:**
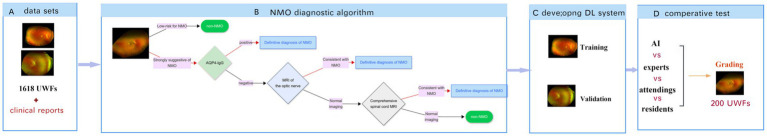
Functional architecture and training pipeline of multimodal AI diagnosis system for NMO. **(A)** Study dataset comprising two modalities: ultrawide-field fundus images (UWFs) and corresponding clinical examination reports. **(B)** Schematic of the NMO diagnostic algorithm workflow. **(C)** Development of the deep learning (DL) system using annotated datasets for training and validation. **(D)** Comparative performance evaluation of MAiDS-NMO for NMO diagnosis against clinical standards.

A panel of six ophthalmologists with different levels of experience (comprising two neuro-ophthalmology specialists, two attending ophthalmologists, and two resident ophthalmologists) were recruited to participated in a diagnostic validation study involving 200 UWFs at the Third Affiliated Hospital of Sun Yat-sen University between January 2022 to April 2024. While the fundus photos were being presented, the AI model identified whether the UWFs depicted NMO. Simultaneously, the ophthalmologists were asked to independently complete the same test as the AI model without access to the clinical examination reports. In the subsequent validation phase, both the MAiDS-NMO and human experts repeated the evaluation with full access to multimodal clinical parameters. The diagnostic performances of the AI model and ophthalmologists were recorded and subjected to comparative statistical analysis. The functional architecture and training pipeline of the AI model and the development process are represented in Figure 4.

## 3 Results

### 3.1 Definitions of NMOSD

The diagnostic criteria for possible NMOSD were based on the following published international consensus diagnostic criteria: (10) an AQP4-IgG-positive serostatus, the presence of at least one of the “core characteristics,” and the exclusion of alternative diagnoses. The diagnostic and exclusion criteria are detailed in Box 1, 2, respectively.

BOX 1 **2015 IPND criteria for NMOSD with AQP4-IgG.**

**Table:** 

Criterion A: Aquaporin 4 (AQP4)-IgG-positive serostatus
Criterion B: (At least one of the following ‘core characteristics’ (which may be the result of one or more clinical attacks)) Clinical evidence for acute opticneuritis Clinical evidence for acute myelitis Clinical evidence for acute area postrema syndrome Clinical evidence for acute brainstem encephalitis other than area postrema syndrome Clinical evidence for acute diencephalitis or symptomatic narcolepsy plus MRI evidence of a periependymal lesion at the level of the third ventricle, or a lesion in the thalamus or hypothalamus Clinical evidence for acute (tel) encephalitis plus MRI evidence of an extensive periependymal lesion at the level of the lateral ventricles, or a large/confluent deep or subcortical white matter lesion (often with gadolinium enhancement), or a longitudinally extensive (≥1/2 of its length), diffuse, heterogeneous or oedematous corpus callosum lesion, or a longitudinally extensive (contiguously from the internal capsule to the cerebral peduncles) corticospinal tract lesion
Criterion C: Exclusion of alternative diagnoses

BOX 2 **Exclusion criteria for NMOSD based on 2015IPND criteria.**

**Table:** 

Criterion A: Clinical Exclusion Criteria
Isolated Syndromes Without Core Features
Pure brainstem symptoms (e.g., isolated vertigo) Isolated cortical syndromes (e.g., seizures) Chronic progressive myelopathy (>3 months progression) Atypical Presentations Peripheral nervous system involvement (e.g., radiculopathy) Non-specific encephalopathy without MRI lesions
Criterion B: Neuroimaging Exclusion Criteria: Location Exclusionary Findings Spinal Cord: Short-segment myelitis (<3 vertebral segments) Optic Nerve: Unilateral involvement with <50% nerve length affected Brain: Dawson’s fingers, ovoid periventricular lesions (MS-like) Orbit: Isolated anterior optic nerve involvement (MOGAD-pattern)
Criterion C: Laboratory Exclusion Criteria Positive MOG-IgG (by cell-based assay, CBA) with compatible clinical/MOGAD features CSF-Specific Oligoclonal Bands (OCBs) (strongly suggestive of MS) Seropositivity for Alternative Diagnoses: Infectious (HIV, syphilis, HTLV-1) Paraneoplastic (anti-CRMP5, anti-amphiphysin) Metabolic (vitamin B12 deficiency, copper deficiency)
Criterion D: Disease-Specific Exclusions Multiple Sclerosis (MS): Meeting 2017 McDonald MRI criteria for dissemination in space/time "Central vein sign” on high-resolution MRI (>40% perivenous lesions) MOG Antibody-Associated Disease (MOGAD): Bilateral optic neuritis with optic disc edema Short-segment or conus-predominant myelitis Other Mimics: Spinal dural arteriovenous fistula (MRI: flow voids, cord edema) Neurosarcoidosis (hilar lymphadenopathy, elevated ACE)

### 3.2 Image datasets and patient clinical characteristics

We established a dataset composed of UWFs and clinical examination reports collected in Guangzhou, Third Affiliated Hospital of Sun Yat-sen University, from January 2022 to April 2024. The demographic and clinical information of the study participants is summarized in Table 1.

**Table 1:** Baseline characteristics of the study participants in the data sets.

Group	NMO			non-NMO		
	Training	Validation	Test	Training	Validation	Test
Participants	219	33	33	798	127	130
Eyes	264	33	33	1,031	127	130
Images	264	33	33	1,031	127	130
Male/female	59/160	7/26	8/25	231/567	38/89	40/90
Ages	38.11 (±11.8)	41.4 (±12.1)	40.5 (±12.7)	39.9 (±13.1)	40.2 (±13.9)	41.2 (±12.5)
AQP4-IgG seropositivity	183	27	29	/	/	/
Optic nerve MRI (consistent with NMO)	171	20	23	/	/	/
Spinal cord MRI (consistent with NMO)	219	33	33	/	/	/
Underlying conditions (N)						
Hypertension	9 (4.1%)	1 (3.0%)	2 (6.0%)	81 (10.2%)	11 (8.6%)	15 (11.5%)
Diabetes	12 (5.4%)	2 (6.0%)	2 (6.0%)	90 (11.3%)	10 (7.8%)	14 (14.7%)
Ocular comorbidities (eyes)						
Glaucoma	6 (2.3%)	2 (6.0%)	3 (9.0%)	51 (4.9%)	10 (7.8%)	14 (10.7%)
Diabetic retinopathy	7 (2.6%)	2 (6.0%)	2 (6.0%)	91 (8.8%)	11 (8.6%)	15 (11.5%)
Retinal detachment	1 (0.3%)	1 (0.3%)	2 (6.0%)	33 (3.2%)	5 (3.9%)	6 (4.6%)
Retinal degeneration	30 (11.4%)	4 (12.1%)	3 (9.0%)	67 (6.5%)	13 (10.2%)	17 (13.0%)
Retinal vein obstruction	8 (3.0%)	2 (6.0%)	1 (3.0%)	27 (2.6%)	2 (1.6%)	4 (3.0%)

First, we developed a model to diagnose possible NMOSD on the basis of 1,618 UWFs from 1,055 individuals seen at the Third Affiliated Hospital of Sun Yat-sen University ophthalmology department. The images were split into training, validation and test datasets at a ratio of 80:10:10 on the basis of a stratified-group-split approach. Among these images, 330 images were associated with clinical records.

### 3.3 Diagnostic performance of MAiDS-NMO based on UWFs captured by SLO

**Figure 5:**
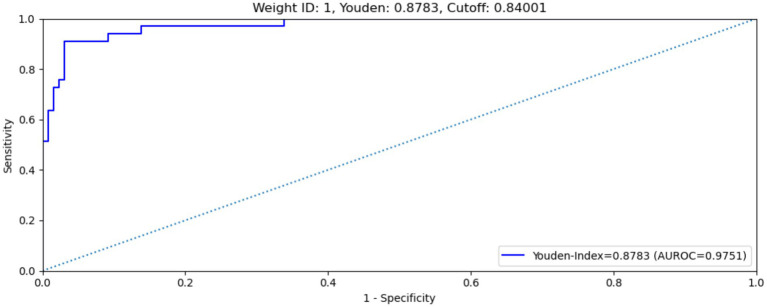
ROC curve of weight no. 1 on test data set. Weight no. 1 shows an AUROC of 0.9751, maximized Youden’s index of 0.8783, sensitivity of 90.9% and specificity of 92.%.

Several performance metrics for selected fine-tuned weights during the training, validation and test processes without use of the clinical results. Weight No. 1 showed the best overall performance among the four selected weights, with an AUC of 0.9751 and a maximum Youden’s index of 0.8783 on the test dataset (Figure 5 shows the ROC curve of the model on the test dataset). This weight was therefore selected as the final weight for the MAiDS-NMO system based on maximization of the Youden index.

**Figure 6:**
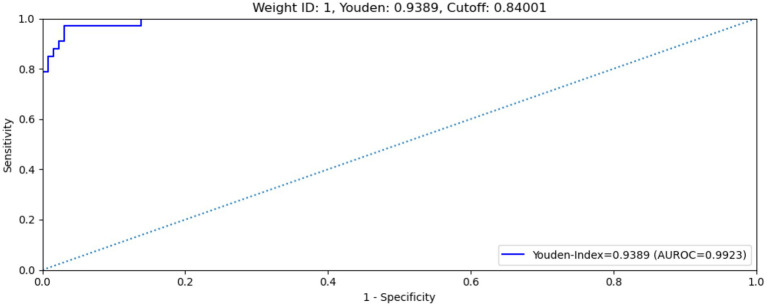
ROC curve of MAiDS-NMO with multimodal input on test data set. The system achieved an AUROC of 0.9923, maximized Youden’s index of 0.9389, sensitivity of 97.0% and specificity of 96.9%.

The MAiDS-NMO system was then evaluated in the test dataset using the optimum cut-off threshold value (0.84) and with multimodal input data. The system achieved an AUC of 0.9923 (ROC curve shown in Figure 6), a maximum Youden index of 0.9389, a sensitivity of 97.0% and a specificity of 96.9%.

The results obtained from five-fold cross-validation provide a more robust and reliable assessment of the MAiDS-NMO system overall predictive performance, mitigating potential biases that may arise from a single training-test split. By employing a systematic approach to data partitioning and iterative validation, this technique ensures the model’s stability and generalizability across diverse data distributions, thereby enhancing its applicability and scalability in real-world clinical settings. The detailed results are presented in Table 2.

**Table 2:** Results of five-fold cross-validation.

Fold ID	Training	Validation	Test
Fold 1	Sensitivity: 87.8%	AUC: 0.971	AUC: 0.9734
		Youden: 0.838	Youden: 0.8249
	Specificity: 89.5%	Sensitivity: 88.1%	Sensitivity: 87.9%
		Specificity: 93.1%	Specificity: 94.6%
Fold 2	Sensitivity: 85.3%	AUC: 0.960	AUC: 0.9774
		Youden: 0.8298	Youden: 0.8331
	Specificity: 89.0%	Sensitivity: 75.0%	Sensitivity: 84.8%
		Specificity: 93.8%	Specificity: 96.9%
Fold 3	Sensitivity: 92.2%	AUC: 0.9311	AUC: 0.9641
		Youden: 0.7208	Youden: 0.8471
	Specificity: 93.4%	Sensitivity: 75.8%	Sensitivity: 78.9%
		Specificity: 94.3%	Specificity: 96.9%
Fold 4	Sensitivity: 92.4%	AUC: 0.9318	AUC: 0.9834
		Youden: 0.726	Youden: 0.8702
	Specificity: 94.3%	Sensitivity: 83.6%	Sensitivity: 84.8%
		Specificity: 87.7%	Specificity: 94.6%
Fold 5	Sensitivity: 88.0%	AUC: 0.9554	AUC: 0.9753
		Youden: 0.782	Youden: 0.8322
	Specificity: 90.7%	Sensitivity: 80.9%	Sensitivity: 84.8%
		Specificity: 90.2%	Specificity: 93.8%

### 3.4 Heatmap

**Figure 7:**
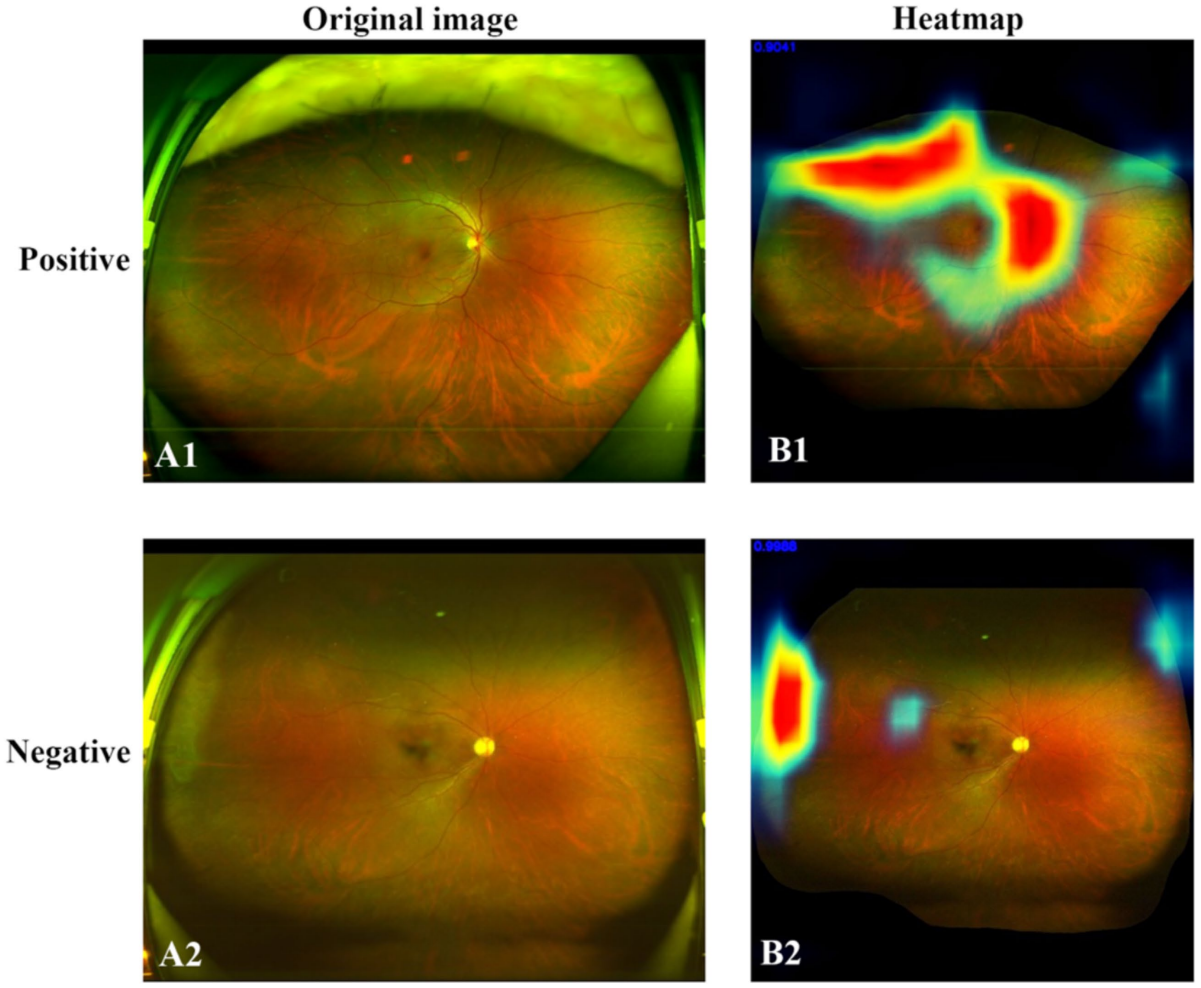
Original SLO image (right) and heatmap (left). **(A1)** Positive case. **(A2)** Negative case. **(B1)** In the NMO-positive case, the areas of high signal intensity closely corresponded to the anatomical distribution of the optic nerve and peripapillary region. **(B2)** In the negative case, no areas of high signal intensity were observed in these anatomical regions.

Heatmaps were generated and superimposed onto the fundus images to visualize the relative contributions of each pixel in predicting the grading for each image. In-NMO-positive cases, the regions exhibiting the highest signals intensity—indicating the most significant influence on the grading prediction—were predominantly localized to the optic nerve and peripapillary areas, aligning with known pathological sites of the disease. In contrast, NMO-negative cases displayed no areas of high signal intensity in the optic nerve and its surrounding regions, suggesting an absence of characteristic NMO-related features. Representative examples of these heatmaps are shown in Figure 7.

### 3.5 Comparative test

**Figure 8:**
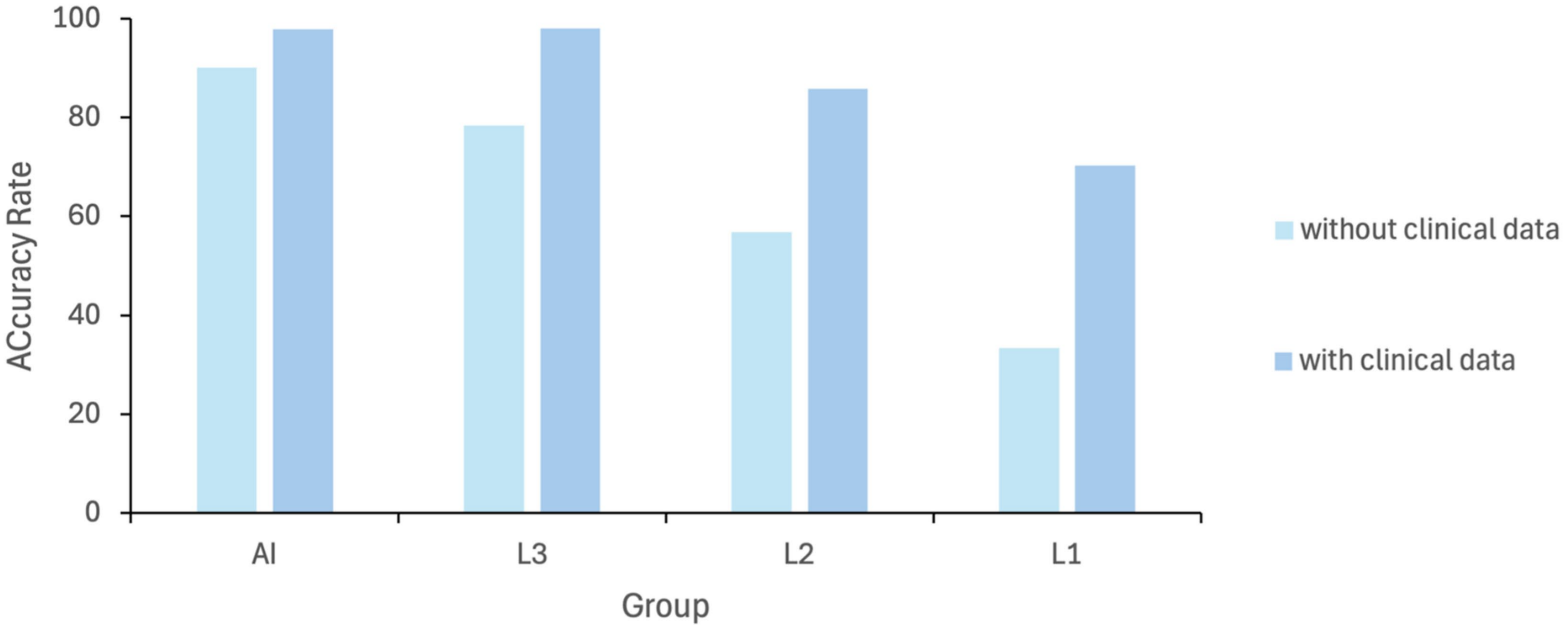
Diagnostic performance comparison (AI vs. ophthalmologists at different levels): AI model for NMO detection; L1: specialists; L2: attendings; L3: residents. L1: senior specialists [>10 years’ experience]; L2: attendings [3–10 years]; L3: residents [<3 years].

To verify the performance of MAiDS-NMO in assessing NMO, a comparative test involving 200 UWFs was conducted between the model and six ophthalmologists. In the initial assessment, the precision rates of the proposed system in the two tests were 90.2 and 97.8% across two tests, demonstrating stable and reliable performance of the neural network. Indicating that the neural network achieved relatively stable training results. In contrast, the accuracy rates of experts, attending physicians, and residents in the first test were 78.3, 56.8, and 33.3%, respectively. However, when provided with clinical information the accuracy rates improved to 98, 85.7, and 70.2%, respectively. Results of the comparative test were shown in Figure 6. These results highlight the potential of MAiDS-NMO in assisting clinical diagnosis, particularly in settings where access to specialized expertise is limited. The detailed results of the comparative test are presented in Figure 8.

## 4 Discussion

NMOSD occurs worldwide and affects individuals of all ethnic backgrounds (24). This severe and widespread disorder has a profound impact on global health, imposing a heavy global burden. Without timely treatment, approximately 50% of NMOSD patients may become disabled and blind, and one-third die within five years of their initial attack (25). Diagnosing NMOSD and monitoring treatment is challenging, burdensome, and clinically time-consuming due to the disease’s variable and often insidious manifestations. Furthermore, access to essential diagnostic tools such as MRI scanning and tests for AQP4-IgG and MOG-IgG is limited in many regions, and some patients are seronegative. Thus, it may be challenging to distinguish NMOSD solely based on brain MRI at disease onset. Misdiagnosis of NMOSD is common, leading to delays in appropriate treatment and potentially resulting in severe or irreversible vision loss. Additionally, NMOSD lesions may be missed if patients are evaluated outside the acute phase or present with atypical clinical symptoms. Differences among ethnic populations, selection bias, and the availability of expert knowledge further complicate accurate diagnosis and analysis.

Screening for NMOSD, coupled with timely referral and treatment, is a widely recognized strategy for preventing blindness. Thus, there is high clinical demand for an efficient and reliable AI model that capable of overcoming the abovementioned obstacles and aiding in the early detection of NMOSD. Such a model could facilitate prompt intervention and improve patient outcomes. Deep learning algorithms have been widely applied in the diagnosis of ophthalmic diseases, demonstrating outstanding diagnostic performance (26, 27). However, to our knowledge, few studies have explored the effectiveness of DL- or AI-based methods in NMOSD diagnosis.

In this study, our AI model achieved exceptional performance in diagnosing NMOSD using UWFs as the input. First, the training dataset was constructed entirely based on the ophthalmologist’s grading of all color fundus images, enabling the development of a DL algorithm with robust accuracy in identifying individuals suspected of having NMOSD at various stages. The model achieved an AUC > 0.97, sensitivity > 90%, and specificity > 92% across all datasets with available data. Second, beyond using UWFs as input, the AI model is well-suited for integrating additional clinical examinations and MRI reports of the optic nerve and spinal cord. This allows for NMOSD subtype classification and disease progression tracking, incorporating multimodal data to enhance predictive performance. Third, the model was trained on both high-quality and low-quality UWFs, ensuring its applicability to images affected by eye movement, media opacities, or other conditions that may obscure image clarity. Lastly, in a comparative test between the model and ophthalmologists, results indicated that while the neural network achieved relatively stable training results, ophthalmologists demonstrated superior diagnostic performance when provided with clinical data for reference.

The shortage of ophthalmologists and neurologists in rural areas has led to delays in NMOSD diagnosis and treatment, leaving many patients without timely medical care. UWFs is noninvasive and user-friendly tool that playing a significant role in telemedicine, proving invaluable in regions with limited access to specialized ophthalmological services. By enabling remote diagnostics and facilitating prompt medical interventions (28), UWF helps bridge healthcare gaps in underserved areas. Our AI-driven algorithm and platform, designed to diagnose NMOSD using UWF images, streamline the diagnostic process without requiring direct involvement from ophthalmologists or neurologists. This innovation has the potential to assist governments in providing more accurate and timely medical support to economically disadvantaged populations, improving healthcare accessibility and outcomes.

There are several limitations to this study. First, the dataset included a relatively small number of patients with NMSOD, largely due to the rarity of the disease in the general population. This limited sample size may have affected the model’s training and its overall predictive performance. Second, all the data were derived exclusively from a single-center, hospital-based Chinese population, without representation from broader community-based or multi-ethnic cohorts. This homogeneity, coupled with the lack of an external validation dataset, restricts the generalizability and external applicability of our findings. Validation using data from multiple centers and more diverse ethnic populations will be essential to confirm the robustness and adaptability of the model. Third, the current application is designed specifically for the early identification or onset prediction of NMOSD but does not address disease progression or recurrence was limited to onset prediction and cannot predict progression, the latter being an essential part of NMOSD management. Despite this limitation, the model still provides clinical value, especially in assisting with the challenging differential diagnosis during the early stages of the disease. Future research with larger, more diverse datasets and longitudinal follow-up will be essential to develop a fully automated and comprehensive deep learning framework capable of both diagnosing and monitoring NMOSD.

## 5 Conclusion

The emergence and advancement of AI have provided new opportunities for improving novel systems and strategies for detecting NMOSD. Our study demonstrated that the proposed DL system exhibits high sensitivity and specificity in identifying NMOSD patients, while also showing potential for predicting disease onset and progression. With improved computing capabilities and advanced database technologies, this model could potentially be developed into a comprehensive virtual screening system for NMOSD in clinical practice.

## Data Availability

The raw data supporting the conclusions of this article will be made available by the authors, without undue reservation.
